# AURP: An AUV-Aided Underwater Routing Protocol for Underwater Acoustic Sensor Networks

**DOI:** 10.3390/s120201827

**Published:** 2012-02-09

**Authors:** Seokhoon Yoon, Abul K. Azad, Hoon Oh, Sunghwan Kim

**Affiliations:** School of Electrical Engineering, University of Ulsan, Ulsan 680-749, Korea; E-Mails: akazad.ju@gmail.com (A.K.A.); hoonoh@ulsan.ac.kr (H.O.)

**Keywords:** underwater sensor networks, *ad hoc* routing, AUVs

## Abstract

Deploying a multi-hop underwater acoustic sensor network (UASN) in a large area brings about new challenges in reliable data transmissions and survivability of network due to the limited underwater communication range/bandwidth and the limited energy of underwater sensor nodes. In order to address those challenges and achieve the objectives of maximization of data delivery ratio and minimization of energy consumption of underwater sensor nodes, this paper proposes a new underwater routing scheme, namely *AURP* (AUV-aided underwater routing protocol), which uses not only heterogeneous acoustic communication channels but also controlled mobility of multiple autonomous underwater vehicles (AUVs). In AURP, the total data transmissions are minimized by using AUVs as relay nodes, which collect sensed data from gateway nodes and then forward to the sink. Moreover, controlled mobility of AUVs makes it possible to apply a short-range high data rate underwater channel for transmissions of a large amount of data. To the best to our knowledge, this work is the first attempt to employ multiple AUVs as relay nodes in a multi-hop UASN to improve the network performance in terms of data delivery ratio and energy consumption. Simulations, which are incorporated with a realistic underwater acoustic communication channel model, are carried out to evaluate the performance of the proposed scheme, and the results indicate that a high delivery ratio and low energy consumption can be achieved.

## 1. Introduction

Underwater acoustic sensor networks (UASN) can provide a cost-effective underwater monitoring solution by tackling problems of traditional ocean monitoring approaches which rely on off-line monitoring [1] or underwater infrastructures [2], where costly underwater optical cables are used for communications between sensors and base stations. For example, UASN can be deployed without significant infrastructures for ocean environmental monitoring [3], seismic monitoring [4], underwater tactical surveillance [5], ecology and habitat monitoring.

However, designing an UASN brings about several new challenges due to the power limitations of underwater sensor (U-sensor) nodes and the unfavorable characteristics of the underwater acoustic channel such as limited bandwidth, multi-path effect, fading, large propagation delay, and high bit error rate [4] (As an example of limited underwater communications, only about 48 Kbps data rate can be achieved for 2 kilometer distance over 23 KHz bandwidth [6], while terrestrial microwave communications can achieve over 100 Mbps for a distance of several kilometers [7]). More specifically, these limitations of the underwater communication make it hard to design a high performance network [8], *i.e.*, a low available data rate, high variation of propagation delay depending on the distance, and a high bit error rate result in low network performance in terms of throughput and delivery ratio [9]. When multi-hop transmissions are required for data delivery in a large scale underwater network, it is obvious that the performance degradation becomes more severe due to the accumulated effects of the communication limitation over multiple forwarding processes. In other words, excessive multi-hop transmissions can lead to a low data delivery ratio because of network congestion and packet errors.

In order to address the problem of the low delivery ratio and to minimize energy consumption of U-sensors, we propose a novel underwater sensor network architecture and routing scheme, namely *AURP* (AUV-aided underwater routing protocol), that use not only heterogeneous acoustic communication channels but also controlled mobility of multiple autonomous underwater vehicles (AUVs). AURP minimizes the total number of data transmissions by using AUVs as relay nodes, which results in a high packet delivery ratio and low energy consumption. Furthermore, by exploiting controlled mobility of AUVs and the fact that AUVs can move closer to other UASN nodes, a short-range high data rate acoustic channel can be applied [10]. As a result, AURP opens a new possibility for an UASN to provide applications that require mass data transmissions, such as multimedia applications.

**Figure 1.**
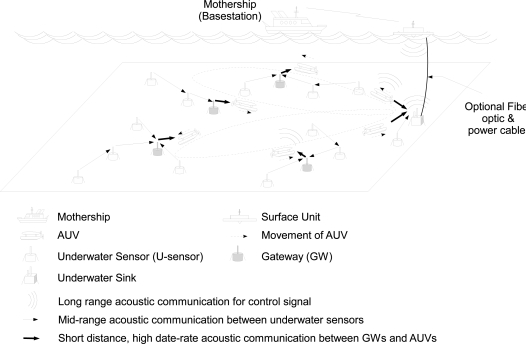
Underwater Acoustic Sensor Network with multiple AUVs.

Figure 1 illustrates the concept of AURP and the network architecture, which consists of U-sensor nodes, gateways (GWs), AUVs, and surface unit/mothership. As shown in Figure 1, in AURP, each U-sensor sends sensed data, using a midrange underwater acoustic channel, to the GWs directly or in a multihop fashion. The GWs forward the aggregated data to an AUV using a short-range high rate acoustic channel when the AUV passes by a short distance from the GWs. When an AUV approaches the underwater sink, it forwards, also using a short-range high rate acoustic channel, collected data to the sink, which will in turn forward the data to the surface unit. AUVs have another acoustic interface for a long distance and low data rate communications to receive control signal and to send urgent data from/to the mothership via a surface unit. The underwater sink and GWs periodically broadcast their interests in receiving data, which are used by U-sensors to choose the next hop node such that the number of hops of transmission is minimized.

To verify the performance of AURP, we perform simulations using NS2 and compared AURP with another routing protocol that is based on Directed Diffusion [11]. According to the simulation results, AURP shows a much higher performance in terms of delivery ratio and energy consumption. Other contributions of this work includes that a realistic underwater communication channel model is incorporated with the simulation to obtain more reliable results, as well as various AUVs’ movement patterns are considered.

It is worth noting that there have been several works that use robotic agents to carry data [12–15]. However, those works focus on scheduling of mobile elements to prevent buffer overflow rather than considering the overall network architecture and the network performance. Furthermore, our work also consider network energy consumption and the critical issues of underwater communication environment which will significantly affect performance of the real world network operation.

The rest of the paper is organized as follows. Section 2 presents problem definitions and model. In Section 3, related work is discussed. Section 4 elaborates our proposed network architecture and routing protocol. In Section 5, the performance of AURP is evaluated by comparing with another routing protocol. Section 6 concludes the paper.

## 2. Problem Definition and Model

In this section, we discuss in more detail the problem under consideration and the system model. Limitations of underwater communications bring challenges when deploying a practical UASN. Unfortunately, those limitations have more significant effects on the network performance when a large scale multi-hop underwater sensor network needs to be deployed. More specifically, packets have a higher possibility of being impaired when they are relayed in a multi-hop fashion due to accumulated effects of the communication limitations. In addition, the signal interference becomes a significant challenge due to the fact that several copies of packets need to be transmitted by a low bandwidth channel. As a result, the network capacity decreases significantly, and many applications (e.g., multimedia data such as snapshots, low rate video, *etc*.) requiring a relatively high data rate transmission cannot be used.

In this work, we aim at tackling those limitations by designing an underwater routing scheme that can scale with a large number of nodes and can reliably deliver a large amount of data. More specifically, our major design objectives include the maximization of the data delivery ratio and the minimization of energy consumed by U-sensor nodes.

In the UASN under consideration, there are a large number of U-sensors that are deployed on the underwater surface. In the UASN under consideration, U-sensors can be stationary or mobile. Stationary U-sensors are anchored with strings to keep the position despite the underwater current. The collected sensed data by U-sensors are sent in a multi-hop fashion to the underwater sink that communicates with the surface unit via a long distance acoustic channel to transmit and receive aggregated data and control signal, respectively. Optionally, the underwater sink can be connected with the surface unit via a fiber optic and power cable in case very high data rate transmission is required. Multiple *AUVs* (Autonomous Underwater Vehicles) are also used to route sensing data from the U-sensors to the sink.

Some U-sensors are equipped with two network interfaces: one is for mid-range communications with other U-sensors, and the other is for short-range and high data rate communications with AUVs. Those sensors with two network interfaces can be *GWs* (gateways), and they communicate with AUVs when AUVs are close enough for the high data rate transmission. The GWs can be pre-determined before deployment or selected by AUVs during the initialization phase. AUVs also have more than one interfaces: one is to communicate with GWs/sink and the other is to communicate with the surface unit to receive motion control signals.

## 3. Related Work

In this section, we discuss the feasibility of our assumptions in underwater communications and networking by pointing out various studies recently carried out in this field.

### 3.1. Underwater Communication and Networking

Underwater communications and networking have been extensively studied in [4,10,16–21]. The study in [16] demonstrated the chronological development of different modulation techniques for underwater communications, and discussed the achievable data rates and transmission ranges. The authors of [10] showed that the data rate of 500 kbps can be achieved between two communicating peers with a distance of 60 m in deep water by using PSK modulation technique. Several challenging issues for underwater networking were discussed in [4] including power consumption behaviors of underwater sensor nodes, and the bizarre characteristics of underwater acoustic communication channel such as limited bandwidth, high level of multi-path effect, fading, doppler spreading, high propagation delay, and high bit error rate. The study in [17] proposed transmitter-based underwater MAC (UW-MAC) protocol combining the properties of both ALOHA and CDMA. The study in [18] has shown that multi-hop underwater communication can be more energy efficient than a single-hop communication in a long distance.

To increase the data rate over the bandwidth-limited acoustic channel, either the bandwidth, or the spectral efficiency in the unit of bits per second per Hertz (b/s/Hz), or both, need to be increased [20,21]. Toward achieving this goal, the studies in [20] showed that MIMO-OFDM technique can easily attain spectral efficiency of 3.5 bits/sec/Hz in one experiment and a data rate of 125.7 kb/s over a bandwidth of 62.5 kHz in another for an acoustic channel of distance 1,500 m.

There also have been studies on alternative underwater communication technologies for short-range, high rate data transmissions [19,22–25]. For example, it was shown in [22] that 1 Gbps data rate can be achieved for the distance of two meters using underwater optical link. In [23], integrating suitable MODEM using omnidirectional optical transceiver, a preliminary design of optical communication system was carried out to realize 10 Mbps data rate between two underwater sensor nodes. Also, in [19], authors not only discussed the short-range, high-bandwidth optical communication link between an AUV and a fixed sensor station, but also proposed underwater communications using low-cost light emitting diodes (LEDs). The authors of [24] studied electric current based underwater communications, and showed that 1 Mbps data rate is achievable for the short distance (e.g., 10 m) communications.

The possible integration of a short-range optical communication technology and long range underwater acoustic communications is studied in [25]. This communication system can be used for an AUV to communicate with fixed underwater sensors with a high data rate, which can justify our assumption of short-range and high rate communications between AUVs and GWs. Those related studies show that our assumptions on short communication range for attaining a large amount of data transmissions is reasonable. Furthermore, the development of alternative underwater communication technologies has enhanced the possibility of a short-range and very high rate data transmission between AUVs and underwater devices.

### 3.2. Underwater Monitoring

In this section, we discuss various underwater monitoring systems and technologies.

#### Infrastructure Based Monitoring

Several infrastructure-based underwater observation projects have been recently performed for long term oceanography studies [26,27]. As a representative example, the Neptune project [26] established an undersea observatory network connected by fiber-optic and power cables, which provide high communication bandwidth and ample power to the observatories for real-time and long-term four dimensional remote oceanographic monitoring. Thirty Neptune nodes are spaced in the sea bed where each node is approximately 100 km apart, which leads to thousands of kilometers of cable length.

Our work is different from those systems in that AURP can be applied without significant underwater infrastructures such as underwater cables.

#### Underwater Acoustic Sensor Networks

Intensive research is going on to discover a suitable framework for underwater sensor networks [10,28–34]. In [28], authors proposed two distinct UASN design structures: the first one resembles the conventional cellular system based on a base station, whereas the second one focuses on decentralized *ad hoc* network with multihop routing. The study in [29] classified the UASN architecture into three categories, among which hybrid architecture is analyzed in [10,30,31]. The authors of [30] studied an energy efficient and delay tolerant sensing model that requires strict time constrains.

In [32], in order to cover a large scale underwater area with minimal power consumption, a hierarchical architecture called tree of wheels (ToW) topology is proposed. To the same direction an architecture named TurtleNet [33] is proposed, which incorporates vertical mobility of the underwater sensor nodes to increase the overall throughput of the network. In TurtleNet, a sensor node performs data transmissions in dual modes; when a node is underwater, it uses short-range acoustic modem for data transfer and upon emerging to the surface, the node uses radio communications.

These works are different from ours in that AURP uses multiple AUVs in order to improve the routing performance.

#### Underwater Monitoring Using AUVs

The studies in [3,35,36] have used a single AUV for the oceanographic study. The authors of [3] deployed one AUV for five hours to survey a 48 km^2^ coral mound field in Florida, USA. In another study [35], one AUV, equipped with various underwater sensors, made 165 dives to survey a distance about 2,500 km for the seafloor dynamics study. Those works have different purposes from our work which focuses on the long term monitoring in a large area using multiple AUVs.

There have been several works that used multiple AUVs for ocean sampling [37–40]. For example, in [37], underwater gliders periodically surface to transmit data to the on-shore base-station using a satellite or RF communication link. Note that those works focus on the control of multiple AUVs that are equipped with underwater sensors and hence act as mobile sensors. In contrast, in AURP, a new underwater acoustic sensor network architecture is considered, where AUVs operate as relay nodes that receive sensed data from underwater sensors and forward to the sink. Moreover, in our work, the network performance and energy consumption of U-sensors are analyzed, which has not been studied in those works.

## 4. AURP (AUV-Aided Underwater Routing Protocol)

In AURP, to route the packets to the sink with a high delivery ratio, U-sensors and GWs cooperate with AUVs, which periodically visit the sink and GWs to deliver the received data during its journey.

There are three possible cases how U-sensors deliver sensed data to the sink node. First, U-sensors send the data to the sink using a direct acoustic link. Secondly, U-sensors send the data to other U-sensors, which will then forward the data to the sink or other U-sensors to deliver the data in a multi-hop fashion. Finally, using a direct acoustic link or multi-hop transmissions, U-sensors send the data to a GW which in turn forwards the data to an approaching AUV, which will deliver the aggregated data to the sink when it passing by the sink. Although the data from the U-sensors can be relayed via AUVs or directly without involving AUVs, U-sensors are unaware of whether or not the data are being relayed by AUVs, *i.e.*, routing data via AUVs is transparent to the U-sensors, which allows U-sensors to operate under a single and consistent network protocol.

In order to determine the path from U-sensors to the sink node or GWs, the sink node and GWs periodically flood *PHE* (Pheromone) messages to the network. Upon receiving *PHE* messages, based on the length of the path (e.g., the number of hops) that those messages have taken, each U-sensor determines the next hop to forward the data, *i.e.*, a U-sensor selects the next hop such that the path length is minimized. A U-sensor also forwards *PHE* messages received from other U-sensors, GWs, or the sink. Rather than forwarding all *PHE* messages that it has received, a U-sensor forwards only *PHE* messages that are sent by its next hop node. As a result, the number of flooded *PHE* message is independent of the number of GWs. In fact, the number of *PHE* message sent is asymptotically *O*(*n*) during a message transmission interval where *n* is the number of U-sensors. Therefore, the network can scale with the number of GWs and the sink.

**Figure 2.**
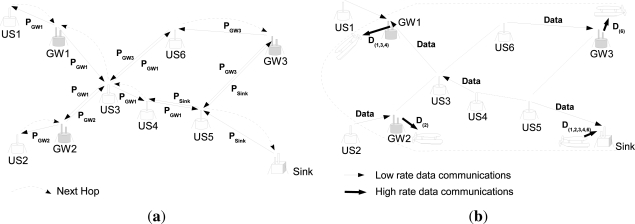
AUV-aided Underwater Routing Protocol: (**a**) Next hop determination based PHE Msg; (**b**) Data forwarding and aggregation.

Figure 2(a) shows an example of determination of the next hop using *PHE* messages. In the example, *US6* (U-sensor 6) received two *PHE* messages, *P*_*GW*1_ and *P*_*GW*3_, each has been generated by *GW*1 and *GW*3, respectively. Since *P*_*GW*3_ has the less value of the number of hops (e.g., 1), *US6* selects *GW*3 as the next hop (the next hop is represented by a dotted line in the example). Then, *US6* forwards *P*_*GW*3_ to its neighboring node, *US3*. *P*_*GW*3_ is discarded by *US3*, since *US3* selects *GW*1 as the next hop based on the hop count information as shown in Figure 2(a). In other words, *PHE* messages are flooded only in a local area.

AUVs move in a pre-determined or dynamically controlled movement fashion. Note that, even though determining the trajectories and the movement pattern of AUVs is not within the scope of this paper, in order to gain insight into the effect of AUVs’ mobility, we analyze the network performance with a various AUVs’ movement patterns and the number of AUVs in Section 5, which assures that having an optimal AUVs’ movement pattern is important to achieve a maximal network performance.

When a GW detects that an AUV is passing by and it is close enough for data transmission, the GW sends data that it has collected from U-sensors to the AUV using a short-range, high data rate acoustic channel. In other words, GWs and AUVs take an advantage of the fact that they can be geographically close to each other, which allows a short-range high rate communication. Note also that the transmissions in a separate acoustic channel do not interfere other transmissions among U-sensors and GWs. The short-range acoustic communications is also used for data transmissions between AUVs and the sink node.

An example of sensing data transmission is illustrated in Figure 2(b). Each U-sensor sends its data to the GW or the sink according to its next hop information. GWs send collected sensing data to the AUV when they become close enough for high rate data transmission. For example, the AUV receives from *GW*1 the data generated by *US1*, *US3*, and *US4*. The AUV forwards the received data from GWs to the sink. Note that *US5* directly transmits its data to the sink according to is next hop information.

Now, we describe the algorithms of each network element in more detail.

### 4.1. Sink Nodes

The sink node broadcasts, at every *s* seconds, to the network *PHE* messages, which will be forwarded and used by U-sensors to determine the next hop to the sink. When the sink node receives beacon signals from an AUV, it initiates negotiation with the AUV to determine the channel for data transmission, the transmission rate, and the modulation scheme. Then, the sink node receives the aggregated data from the AUV using the negotiated short-range/high-rate channel. The sink node also receives data from U-sensors that have generated the data or are relaying data generated by other U-sensors. A sink node discards PHE messages from GWs. It is worth noting that AURP can be readily extended for an architecture with multiple sink nodes.

### 4.2. Underwater Sensor Nodes

U-sensor nodes collect data using equipped sensors and periodically send the data to the sink. U-sensors determine the next hop to forward the data packet based on the information in the *PHE* messages that they have received from the sink or GWs. More specifically, when a U-sensor receives a *PHE* message from a GW or the sink, it stores the message in *DET_NEXT_HOP_QUEUE*. The U-sensor also maintains a timer, *DNH _TIMER*. When the timer expires, the U-sensor examines the travel time of the *PHE* message and the number of hops that the *PHE* message passed from GWs or the sink. Based on the information, the U-sensor determines the next hop that will forward the sensed data to a GW or the sink, such that the data can be delivered to GWs/sink with the minimum path length and delay.

Other routing metrics such as ETX (Expected Transmission Count) and ETT (Expected Transmission Time) can be also applied for path selection.

### 4.3. Gateway Nodes

Similarly to the sink node, GW nodes periodically flood *PHE* messages that will be used by U-sensors. When a GW receives data packets destined to the sink from the U-sensors, it stores them in a queue until it hears a beacon signal from an AUV. Upon reception of a beacon signal from an AUV, the GW and the AUV negotiate on the communication channel and send the stored data to the AUV using the selected channel.

If a GW node receives *PHE* messages generated by other GWs or the sink node, it simply discards the messages.

The GWs can be pre-determined or dynamically selected by AUVs. In case the positions of GWs are pre-determined, more optimal trajectories of AUVs can be applied and dedicated and more powerful U-sensor nodes can be used as GWs.

### 4.4. AUVs

Multiple AUVs move around the survey area to collect data from GWs and forward it to the sink when it passes by the sink. Each AUV periodically broadcasts a beacon signal to show its presence to GW nodes and the sink node. AUVs communicate with GWs and the sink node using a separate short-range, high rate data channel. As a result, AUVs, GWs, and the sink form a separate collision domain for the medium access from the remaining network (e.g., U-sensors and GWs), which significantly reduce the packet collisions in a multi-hop network.

If an AUV has failed (e.g., due to communication or mechanical malfunction) the data it carries can be lost. One possible way of recovering from this loss is that the GW keeps the data, after it sends data to an AUV, until it receives acknowledgement (ACK) from sink via the AUV at the next AUV’s visit. If the GW does not receive ACK from the AUV, it assumes that the data was lost and retransmits it to the AUV. Another point to be considered is how fast the failure can be detected to minimize the recovery delay. Fast failure detection can be achieved since the mothership usually keeps track of the AUV.

## 5. Performance Study

In this section, we first describe underwater acoustic channel model that is used for the simulation study. Then, we discuss the design of AUVs’ movement pattern. Finally, we elaborate the simulation setups and results, and then analyze the performance of the AURP.

### 5.1. Underwater Acoustic Channel Model

As the underwater environment is remarkably different from its terrestrial counterpart [4,35], the existing wireless modules cannot be deployed without specific re-engineering. The internodal distance as well as the link orientation of the underwater network highly affects the underwater channel characteristics [35]. Thus the underwater channel is attributed as the combination of the worst aspect of terrestrial mobile for poor quality and satellite radio channel for high latency [8]. Therefore, it is crucial to accurately emulate underwater channel to warrant the outcome of a simulation process. A lot of research in this context [41,42] have tried to include the impact of loss and delay of the underwater communication into their acoustic channel design.

In order to accurately emulate the underwater communication environment for simulation, we have used an underwater acoustic channel model designed in [43]. In [43], the absorption at a given frequency is approximated based on the Thorp’s approximation shown in [44]. To estimate the total attenuation and eventually obtain the SNR that is used by the NS2 simulator, the model in [43] also incorporates spreading loss and various underwater noises, such as turbulence, shipping, wind, and thermal noises.

The authors of [43] also modeled another important underwater communication factor, the underwater acoustic propagation speed based on [45]. Therefore, in our simulation, the propagation speed *s_p_* in meter per seconds is calculated according to the model shown in [43,45], which is as follows

$$s_{p}=1449.05+45.7t-5.21t^{2}+0.23t^{3}+(1.333-0.126t+0.009t^{2})\;(S-35)+16.3z+0.18z^{2}$$

where *t*, *z*, and *S* represents one tenth of the water temperature, the depth and the salinity of the water, respectively.

For example, we obtain approximately 0.33 s as a propagation delay over a distance of 500 m at a depth of 2,000 m from the surface level.

### 5.2. AUV Movement Pattern

In order to gain an insight of the effects of AUVs’ movement on the performance of underwater network, we design simple yet practical AUV movement patterns, where AUVs move along an elliptical trajectory. More specifically an AUV trajectory is defined by an equation of ellipse, $\frac{{\left(x-x_{c}\right)}^{2}}{a^{2}}+\frac{{\left(y-y_{c}\right)}^{2}}{b^{2}}=1$, where *a*, *b*, *x_c_*, and *y_c_* are real numbers. Another variable *θ* is used to denote the angle of rotation from the positive Y-axis.

Note that the network simulator NS2 only supports a linear movement between two positions at a given constant speed. In order to make the designed AUVs’ movement pattern work with the NS2 simulator, an elliptical trajectory is partitioned into a large number of small line segments that are represented by a list of (*x*, *y*) positions. Assuming an ellipse is centered at (0, 0) and *θ* = 0 in order to facilitate the discussion, the position of AUV at time *t*, (*x_t_*, *y_t_*), is obtained by using following equation.

(1) $$\frac{x_{t}^{2}}{a^{2}}+\frac{y_{t}^{2}}{b^{2}}-1-\sqrt{{\left(x_{t}-x_{t-1}\right)}^{2}+{\left(y_{t}-y_{t-1}\right)}^{2}}+\Delta t*v=0$$

where (*x*_*t*−1_, *y*_*t*−1_), Δ*t*, and *v* are the previous position of the AUV at time *t* − 1, the unit time in the NS2 mobility trace, and the average speed of AUV respectively. Note that between two solutions of Eq. (1), only one is selected as the next position that corresponds to the current move direction of the AUV.

**Figure 3.**
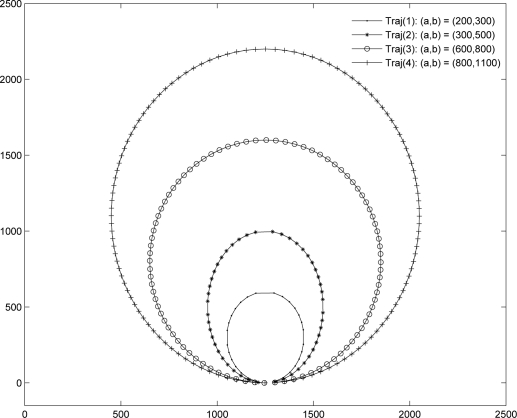
Elliptical trajectories of one AUV.

**Figure 4.**
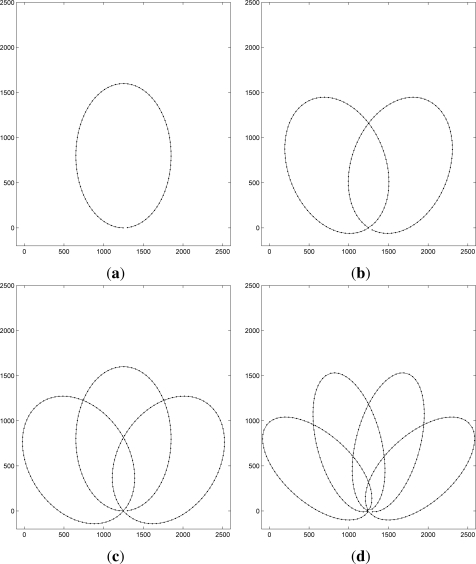
Trajectories of multiple AUVs.

Figures 3 and 4 show the AUVs’ movement pattern in an area of 25,000 m × 25,000 m used for the performance study. In Figure 3, one AUV is considered with four different parameter sets for elliptical trajectories, *Traj*(*i*) where 1 ≤ *i* ≤ 4. The sink node is assumed to be located at the bottom point of the each path. In Figure 4, multiple AUVs’ trajectories are shown. Let each movement pattern be denoted by (*a*, *b*, *θ*). Then, Figure 4(a) shows the trajectory with one AUV, where (*a*, *b*, *θ*) = (600, 800, 0). Two AUV case is shown Figure 4(b), where (*a*, *b*, *θ*) = {(500, 800, $\frac{\pi }{6}$), (500, 800, − $\frac{\pi }{6}$)}. Figure 4(c) show trajectories of three AUVs, where (*a*, *b*, *θ*) = {(400, 800, $\frac{\pi }{4}$), (400, 800, 0), (400, 800, − $\frac{\pi }{4}$)}. Finally, four AUVs’ trajectories are shown in Figure 4(d), where (*a*, *b*, *θ*) = {(300, 800, $\frac{3\pi }{10}$), (300, 800, − $\frac{\pi }{10}$), (300, 800, − $\frac{\pi }{10}$), (300, 800, − $\frac{3\pi }{10}$)}.

It is worthwhile to note that the trajectories of AUVs can be better optimized with the topology information, in particular, the positions of GWs. More specifically, both positions of the GWs and the trajectories of AUVs can be calculated before deployment such that the performance of the underwater sensors network is maximized.

### 5.3. Simulation Results and Analysis

In order to test the routing scheme proposed in Section 4, we use NS2 simulator and also incorporate the underwater channel model developed by the authors of [43]. U-sensors and AUVs are deployed within a 2,500 m × 2,500 m underwater region. The range of numbers of U-sensors and AUV are (20,80) and (1,4), respectively. The default value of the number of U-sensors is set to 50. The depth of water is assumed to be 2,000 m, which leads to around 0.16 s propagation delay over a distance of 250 m. To obtain more practical simulation results, the specification of a commercial acoustic modem, *UWM2200* manufactured by LinkQuest Inc. [46], is used. For example, the transmit and receive power of the acoustic interface are set to 6 W and 1 W respectively, and the maximum data rate of the acoustic interface is set to 14 Kbps. It is assumed that each U-sensor periodically (every 30 s) sends data of 100 Bytes to the sink. GWs and the sink broadcast *PHE* messages at every 150 and 60 s, respectively. AUVs broadcast a beacon signal at every 20 s, and it is assumed that AUVs can be periodically recharged or replaced during the underwater monitoring operation. Thus, in this work, we assume that only energy consumption of U-sensors affect the network lifetime. The average speed of AUVs set to 9.7 knots, which can be achieved by latest AUVs (e.g., AUV developed in [47] can move with a speed of 15 knots).

In order to verify AURP, we compare it with a sensor network routing protocol, referred as *Grad* hereafter, which is based on interests and gradients used in Directed Diffusion [11]. In *Grad*, The sink periodically floods *Interests* to the network, which results in formation of *Gradient* in the opposite direction to the flow of the *Interests*. The source nodes and relaying nodes use the *Gradient* to deliver data.

**Figure 5.**
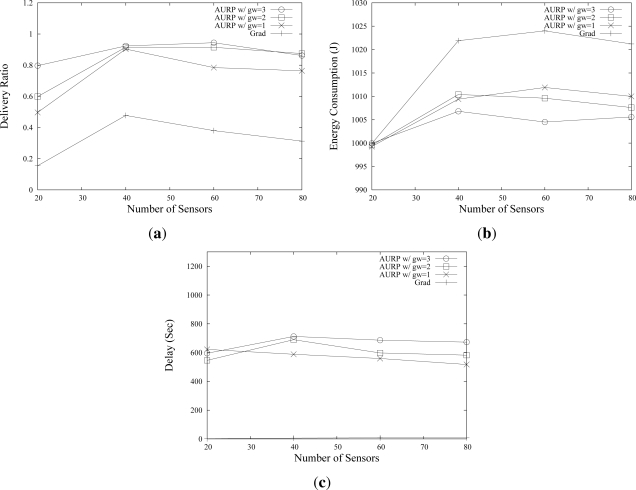
Effects of number of U-sensors on the network performance.

Figure 5 shows the effect of the number of U-sensors on the network performance. As many as 50 U-sensors are randomly deployed in the area, and one AUV is used. AURP with different numbers of GWs, from 1 to 3, is compared with *Grad*. As shown in Figure 5(a), AURP and *Grad* show a low delivery ratio when the number of U-sensors is 20. The reason of a low delivery ratio with 20 U-sensors is that there are not enough U-sensors to form a connected network. In a partitioned network, as shown in Figure 5(a), *Grad* shows a very poor performance since a large portion of the sensing data can not be relayed to the sink. Meanwhile, AURP can achieve a relatively higher delivery ratio even in a partitioned network. When there are 40 U-sensors, AURP achieves over 0.9 delivery ratio, while *Grad* achieves only about 0.5 delivery ratio. As the number of U-sensors grows, the delivery ratios of AURP and *Grad* decrease, since the network becomes congested due to the high network traffic. Note that the delivery ratio of AURP is much higher than *Grad* even in a dense network. For instance, with 80 U-sensors, AURP shows the delivery ratio in the range of (0.78, 0.88) depending on the number of GW, which is higher than twice of *Grad* case. The results shown in Figure 5(a) also implies that deploying more GWs can improve the delivery ratio.

In a network using AURP, the U-sensors consume less energy over different number of U-sensors than U-sensors in a network using *Grad*, as shown in Figure 5(b). The gap of energy consumption becomes greater as the number of U-sensors increases. Note that, in this work, the energy consumption of U-sensors is of interest, hence only U-sensors’ consumed energy is collected.

As shown in Figure 5(c), the packet delay in AURP is much higher than that of *Grad*, since a large portion of packets are carried by AUVs to be delivered to the sink. In other words, the packet delay mainly depends on the travel distance and the speed of AUV. Even though the delay can be reduced by optimizing the trajectory and increasing the speed of AUVs, the *store-carry-forward* process will lead to an inevitable packet delay. Therefore, the proposed network architecture will be suitable for an underwater monitoring system that requires high delivery ratio, low energy consumption, and allows the packet delay of several hundred seconds. Although this delay may pose a limitation in applying the network to some military/tactical applications that requires strictly real time data transmissions, the proposed architecture can be used for most applications that can tolerate several minute delay yet require high rate and reliable data transmissions. The packet delay results in other scenarios are similar due to the fact that the packet delay mostly depends on the AUVs’s speed and trajectories rather than network situation. Therefore, from now on we focus on the data delivery ratio and energy consumption as performance metrics.

**Figure 6.**
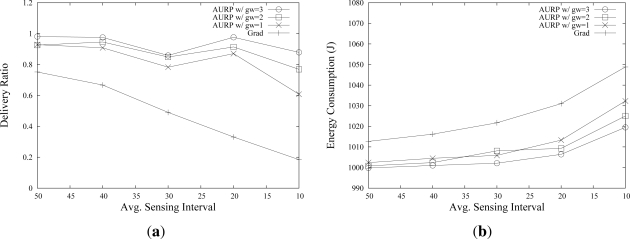
Effects of Network Loads on the network performance.

Figure 6 shows the effect of the network load on the performance. The × axis represents the sensing intervals from 50 s to 10 s. As shown in Figure 6(a), AURP shows a much higher data delivery ratio than *Grad* in all cases. The figure also shows that the performance is improved with a larger number of GWs. Moreover, the difference of delivery ratio values becomes larger when the network load is high. As shown in Figure 6(a), when the sensing interval is 10 s, the data delivery ratio of AURP with three GWs is around 0.9, while that of *Grad* is less than 0.2. It is because, when the network load is high, the transmissions in *Grad* experience network congestion which results in a high level of packet collision. On the other hand, in AURP, the increase of the number transmissions is much lower than that of *Grad* due to less number of hops between U-sensors and the sink. The total energy consumption of the U-sensors using AURP is lower than those with *Grad*. As shown in Figure 6(b), *Grad* based U-sensors consume more energy for data transmission and reception than U-sensors using AURP. This result also comes from the fact that U-sensors using AURP transmit less packets.

**Figure 7.**
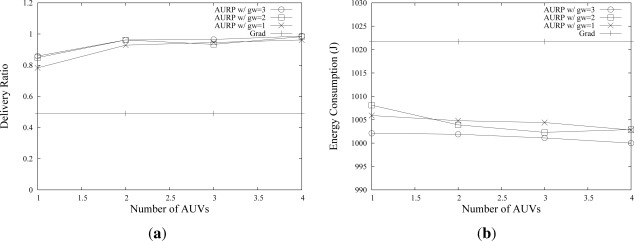
Effects of Number of AUVs on the network performance.

A higher performance of AURP can be achieved by using more than one AUV. Figure 7 shows the effect of multiple AUVs on the delivery ratio and energy consumption. As the number of AUV increases, a higher delivery ratio can be achieved. For example, AURP achieves 0.97 delivery ratio when (#AUVs, #GWs) = (3,3), while it shows 0.85 delivery ratio when (#AUVs, #GWs) = (1,3) as shown in Figure 7(a). Note that *#GWs* represents the number of GWs for each AUV; if (#AUVs, #GWs) = (3,3), then there are 9 GWs in total. *Grad* has a constant value, approximately 0.5, over the simulations since it does not use AUVs. The number of AUVs also has a positive effect on the energy consumption. As shown in Figure 7(b), the U-sensors use less energy when there are more AUVs, since U-sensors can send data to AUVs with a shorter path.

**Figure 8.**
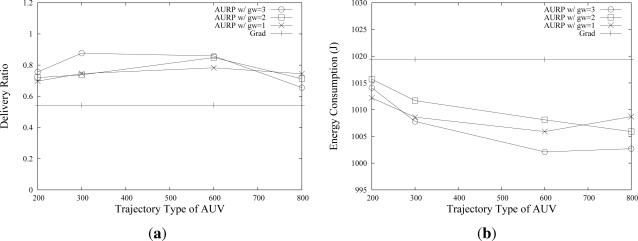
Effects of AUV Trajectories on the network performance.

The trajectories that AUVs follow also affect the network performance as shown in Figure 8. Four trajectories, *Traj*_1_,…, *Traj*_4_, shown in Figure 3 are used. The X-axis represents the value of *a* shown in Figure 3. As shown in Figure 8(a), *Traj*_2_ and *Traj*_3_ show a better data delivery ratio. This is because the number of transmissions due to multihop relay becomes larger when an AUV follows a too small or a too large trajectory, which implies that selecting the trajectory of AUVs is also important to maximize the network performance.

## 6. Concluding Remarks

In this paper, we have proposed an underwater routing scheme, namely *AURP* (AUV-aided underwater routing protocol), to address the problem of the low delivery ratio and high energy consumption in a multi-hop underwater acoustic sensor network (UASN). To achieve a high data delivery ratio and low energy consumption, AURP exploits controlled mobility of multiple autonomous underwater vehicles (AUVs) as well as heterogeneous acoustic communication channels.

In order to verify the proposed scheme, we have incorporated a realistic underwater channel model and various AUVs’ movement patterns with NS2 simulators. The simulation results have shown that AURP can outperform other routing protocols in terms of the delivery ratio and energy consumption, and is a good candidate for many practical applications that require mass data transmissions with a high reliability.
